# Seroprevalence and Risk Factors for Bovine Coronavirus Infection among Dairy Cattle and Water Buffalo in Campania Region, Southern Italy

**DOI:** 10.3390/ani13050772

**Published:** 2023-02-21

**Authors:** Gianmarco Ferrara, Valentina Iovane, Elvira Improda, Giuseppe Iovane, Ugo Pagnini, Serena Montagnaro

**Affiliations:** 1 Department of Veterinary Medicine and Animal Productions, University of Naples Federico II, Via Delpino 1, 80137 Naples, Italy; 2 Department of Agricultural Sciences, University of Naples Federico II, 80055 Naples, Italy

**Keywords:** BCoV, betacoronavirus, serosurveillance

## Abstract

**Simple Summary:**

Since there is currently little information on the distribution of bovine coronavirus (BCoV) in Italy, we determined its seroprevalence among cattle and water buffalo in the Campania region of southern Italy. We found very high seroprevalence values of 30.8% and 76% at individual and herd levels, respectively. In both cattle and water buffalo, species, age, and origin were common risk factors that were positively associated with antibody detection. Only in water buffalo was a higher sero-prevalence observed in animals cohabiting with cattle, indicating that this practice is incorrect. To the best of our knowledge, this is the first BCoV serological survey performed in southern Italy as well as the first description of BCoV antibody detection in water buffalo in Italy.

**Abstract:**

Cattle and water buffalo are the main livestock species that are raised in the Campania region, southern Italy, and they contribute significantly to the regional rural economy. Currently there are limited data on the prevalence of relevant impact infections, such as bovine coronavirus (BCov), an RNA virus that causes acute enteric and respiratory disease. Although these diseases are described primarily in cattle, there have been reports of spillovers to other ruminants, including water buffalo. Here, we determined the seroprevalence of BCoV in cattle and water buffalo in the Campania region of southern Italy. An overall seroprevalence of 30.8% was determined after testing 720 sampled animals with a commercial enzyme-linked immunosorbent assay. A risk factor analysis revealed that the seropositivity rates in cattle (49.2%) were higher than in water buffalo (5.3%). In addition, higher seroprevalence rates were observed in older and purchased animals. In cattle, housing type and location were not associated with higher seroprevalence. The presence of BCoV antibodies in water buffalo was associated with the practice of co-inhabiting with cattle, demonstrating that this practice is incorrect and promotes the transmission of pathogens between different species. Our study found a considerable seroprevalence, which is consistent with previous research from other countries. Our results provide information on the widespread distribution of this pathogen as well as the risk factors that are involved in its transmission. This information could be useful in the control and surveillance of this infection.

## 1. Introduction

The family of coronaviruses (CoVs) includes several positive-sense and single-stranded RNA viruses that commonly infect wild and domestic animals, as well as humans [1]. In addition to the well-known human CoVs (including severe acute respiratory syndrome-related coronavirus, Middle East respiratory syndrome-related coronavirus, and severe acute respiratory syndrome coronavirus 2), the genus Betacoronavirus lists also bovine coronavirus (BCoV), which is closely related to the above mentioned CoVs and belongs to the order Nidovirales, family Coronaviridae, subfamily Orthocoronavirinae [2]. BCoV RNA includes five structural proteins: the spike, the nucleocapsid protein, the haemagglutinin esterase, the integral membrane, and the envelope protein. BCoV was first isolated in respiratory samples by Mebus et al. [3] in 1972, in the United States and represents a primary enteric and respiratory pathogen that is widespread throughout the world. Namely, this virus causes severe gastroenteric syndrome (namely calf diarrhea and winter dysentery in adults, although it can also occur during warmer months) and plays an important role in the bovine respiratory disease complex (BRDC) [4,5,6,7]. It represents a major health problem in calves and is associated with reduced growth performance and increased mortality, resulting in important economic losses for the livestock industry [4,8]. BCoV is frequently involved in coinfections; indeed, during respiratory disease, it is frequently identified with a variety of pathogens such as parainfluenza virus-3 (PI-3), bovine herpesvirus-1 (BHV-1), bovine viral diarrhea virus (BVDV), *Pasteurella* spp., and particularly bovine respiratory syncytial virus (BRSV). During enteric disease, rotavirus (BRV), *Escherichia coli*, *Eimeria* spp., *Cryptosporidium* spp., and other pathogens are frequently found [4]. The diagnosis of BCoV infection is based on nucleic acid detection via reverse transcription polymerase chain reaction (RT-PCR) and real-time quantitative polymerase chain reaction (real-time qRT-PCR) using nasal swab or fecal samples as a matrix, whose sensitivity increases when performed during acute stages of infection. Serological assays, such as the enzyme-linked immunosorbent assay (ELISA), virus neutralization (VTN), and immunofluorescence, are also available to detect specific antibodies. ELISA is usually preferred for intra-herd epidemiological surveys because of its rapidity and applicability for large samples (besides being sensitive and specific). These assays mainly detect antibodies against the dominant epitope (the subunit S1 of the spike protein), whose production begins 7–14 days after the infection [4]. Furthermore, the interest in this virus is increasing due to the susceptibility of ruminants to human coronavirus infection, as well as the potential recombination mechanisms for possible CoV dual infections in a single animal or human [1,9].

In the last decade, several bovine-like coronaviruses have been identified as potential agents of enteric and/or respiratory diseases in a variety of ruminants, suggesting cross-species transmission and the ability to overcome interspecies barriers [10]. These viruses share genomic and antigenic features with BCoV and are widely considered to be host-range variants of BCoV that have emerged from the continuous evolution of the virus. They are inadvertently transmitted to other ruminants mainly by the fecal-oral routes [11,12]. Bovine-like CoVs have been described in domestic ruminants (water buffalo, sheep, goats, llamas, alpacas, and camels); wild ruminants (deer, bison); zoo ruminants (antelopes, giraffes); and even non-ruminants such as dogs and humans [10,13,14,15,16,17].

BCoV and bovine-like coronaviruses have been detected on all continents, and seroprevalence studies have shown high prevalence at both animal and herd levels [1]. However, studies on the prevalence of BCoV and associated risk factors are limited in Europe, and to date no study has been conducted in Italy, although descriptions of the disease in different animal species are regularly reported [18,19,20]. This study focused on the seroprevalence of BCoV infection and assessed its associated risk factors among dairy cattle and water buffalo in the Campania region, southern Italy.

## 2. Materials and Methods

### 2.1. Sampling and Study Area

The current study was carried out in Campania (410000000 N–143000000 E), a region in southern Italy with the largest water buffalo population. There are 158,000 cattle and 306,000 water buffalo that are raised in this area (Banca Dati Nazionale dell’ Anagrafe Zootecnica, https://www.vetinfo.it/j6_statistiche/, accessed on 1 November 2022). Given the lack of recent surveys in the study area, we decided to assume an expected prevalence of 0.5 (i.e., 50% for cattle and 25% for water buffalo which is infected by BCoV-like coronaviruses), an absolute precision of 5%, and a 95% confidence interval [21]. Thrusfield’s formula was used to calculate the sample size, which was as follows:

n = Z^2^ × P(1 − P)/d^2^

where Z = 1.96 for a confidence level of 95%, P = expected prevalence, d = 0.05 accepted error, and n = sample size. Sampling procedures started in October 2020 and were completed in April 2021, coinciding with blood collection conducted for previous studies [22]. The study area included 33 different districts and a total of 22 cattle farms and 24 water buffalo farms (randomly selected) distributed in four provinces (at least 12 animals per farm were sampled). Only unvaccinated farms were sampled, and 419 samples from dairy cows and 301 samples from water buffalo were randomly collected. A vacutainer was used to collect blood samples from the tail vein. After each sample was centrifuged at 1000 *g* for 10 min, aliquots were stored at −20 °C until they were assayed.

### 2.2. Antibody Detection with Commercial Indirect Enzyme-Linked Immunosorbent Assay (ELISA) (SVANOVIR BCV-Ab)

Each sample was tested for BCoV antibodies (IgG) using the “SVANOVIR BCV-Ab” ELISA Test Kit (Boeringer Ingelheim Svanova, Uppsala, Sweden), following the manufacturer’s instructions. This assay was already used in other work described in the literature, as well as during the control program against this disease that was carried out in Norway [23]. In a summary, diluted sera were deposited into the plate and incubated for one hour. Following three washes, conjugate antibody was added and incubated for an additional hour. After 15 min, the substrate solution was added, followed by the stop solution. The optical density was then measured at 450 nm with a spectrophotometer (Thermo Scientific, Carlsbad, CA, USA), and the cut-off value that distinguishes between positive and negative samples was calculated. A map representing the spatial distribution of positive foci was created using Epi Info (EPI Info™ software version 7.2.5.0, Atlanta, GE, USA).

### 2.3. Statistical Analysis

By dividing the number of positive bovines by the overall number of bovines that were tested, prevalence was expressed at the animal level. Risk factor analysis was conducted using data that were gathered during sample selection. For cattle, information about location, housing, age, and origin was evaluated, whereas for water buffalo, information about location, age, origin, and coexistence with cattle was evaluated. Chi-square statistics were used in univariate analysis at the animal level to determine risk factors for BCoV positivity (expressed as binary variables). A *p*-value of 0.05 or less was considered significant. The statistical analysis was performed using MedCalc Statistical Software, version 16.4.3 (MedCalc Software, Ostend, Belgium; www.medcalc.org, accessed on 5 November 2022, and JMP version 14.1.0 (SAS Institute Inc., Cary, NC, USA).

## 3. Results

A total of 720 samples from 46 farms were tested for the detection of specific antibodies against BCoV (419 cattle and 301 water buffalo). Among them, 134 had less than twenty-four months (18.6%) and 586 had more (81.4%). A total of 381 animals were born on the farm, whereas 339 were purchased from other farms. The distribution among different provinces was as follows for bovine: 26.7% Avellino (112/419), 23.15% Benevento (97/419), 27% Caserta (113/419), and 23.15% Salerno (97/419). Among cattle, 27.7% were partly grazed, and the remaining part were bred to be stall-fed. Water buffalo were sampled in the two provinces where this species is mainly raised, Caserta (141/301) and Salerno (160/301). Among them, 29.7% co-inhabited with cattle. Supplementary Table S1 summarizes specific descriptive information about the collected data.

**Figure 1:**
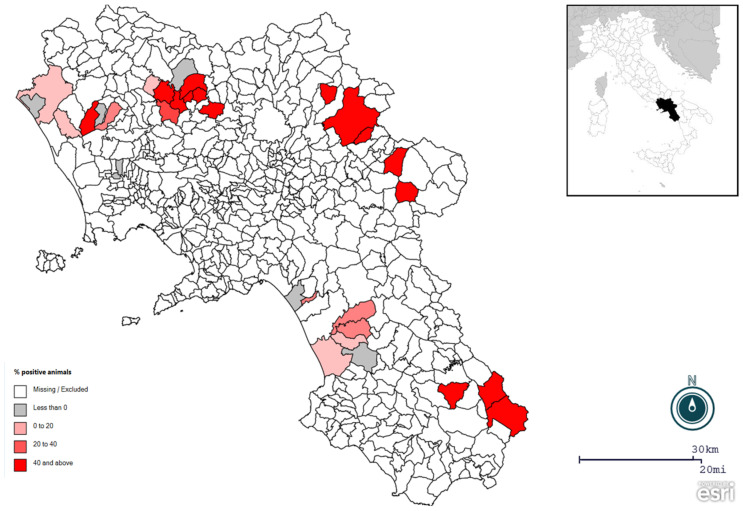
Spatial distribution of positive herds for bovine coronavirus (BCoV).

Our results showed an overall seroprevalence of 30.8% (222/720; CI 95% 27.5–34.2) at the animal level. At the herd level, prevalence was 76% (35/46; CI 95% 63.7–88.4), with 100% (22/22) in cattle and 54.2% (13/24) in water buffalo, with only 11 out of 46 herds having no positive animals. A summary of the results is shown in Table 1 while the wide distribution of positive cattle and buffalo herds is provided in Figure 1.

Seroprevalence of BCoV varied significantly between species (Table 1), with water buffalo having a seropositivity of 5.3% and cattle having a significantly higher seroprevalence of 49.2% (*p* < 0.001). Univariate analysis of common risk factors revealed that age and origin were significantly associated with higher seroprevalence. Seroprevalence was higher in animals that were older than 24 months (34.6%) and in purchased animals, which had a higher chance of being positive when compared to farm-born animals (42.5%).

As the characteristics of husbandry (and consequently the risk factor involved in the transmission of this disease) differ significantly between the two species, risk analysis was conducted separately for some factors (location and type of housing in cattle and location and cohabitation with cattle in water buffalo, as shown by Table 2 and Table 3). The seroprevalence of BCoV in the selected provinces of Avellino, Benevento, Salerno, and Caserta were 43.7%, 54.6%, 55.6%, and 44.2%, respectively. The results showed negligible and non-significant differences between the different provinces (e.g., Salerno and Avellino). Also, location was not a significant risk factor for buffalo (*p* = 0.79), while cohabitation with cattle was significantly associated with higher BCoV prevalence (*p* = 0.003). There were no differences between cattle that were partially grazed (48.3%) and those that were raised for stalling (49.5%) (*p* = 0.908), nor were there any differences between the different locations. 

## 4. Discussion

Despite the knowledge that BCoV is a pathogen with a significant economic impact that has been studied for potential recombination mechanisms that allow it to overcome the species barrier, there are few data in the literature on its spread, particularly in Europe [10]. In Italy, both enteric and respiratory outbreaks are commonly reported, but there is still a lack of information on BCoV spread [18,19,20].

We investigated the seroprevalence of BCoV among cattle and water buffalo in the Campania region of southern Italy and observed a very high number of seropositive animals (30.8% seroprevalence at the animal level). Since BCoV is a widespread infection and within-herd transmission is usually rapid, this result was expected and is consistent with results that were obtained in other countries when apparently healthy animals were tested. In Norway, for example, BCoV antibodies were found in 1014 of 1347 herds in a study using an ELISA test on tank milk [24]. Also in Norway, a large-scale survey of respiratory infections in 2009 found a seroprevalence of 39.3% in calves and 80.7% at the herd level [25]. Further studies, using similar approaches in milk samples, reported a herd seroprevalence that was higher than 70% in two different surveys in Sweden, confirming the strong interest of Scandinavian countries in this infection [26,27]. In Finland, surveillance against this pathogen that was performed on the cattle population also resulted in 89% positive herds and 38% positive animals [28]. Other studies that were published in the literature on different continents found an individual prevalence of 98% in Thailand and 55% in Ghana [29,30]. A recent meta-analysis study that was carried out in China found 53.3% seroprevalence [31]. The data presented are the result of similar epidemiological scenarios, although the reported prevalence values (which are generally very high) may vary depending on other factors such as the approach, the assay that was used, sampling, etc.

In univariate analysis, our results suggest a considerable difference in BCoV exposure between cattle and water buffalo. Similarly, several works that were performed on different continents suggest lower seropositivity in hosts other than cattle, namely small ruminants and wild ruminants [29,32,33]. This aspect proposes that natural infections are common in different hosts, while the difference in seroprevalence may be explained by the different tropism of BCoV for cattle and water buffalo. Furthermore, water buffalo are susceptible to infections with bovine-like coronaviruses such as BuCoV, which, may have different transmission dynamics despite high genetic similarity (up to 99%) with BCoV [11,34,35,36]. Another hypothesis concerns the ineffectiveness of the assay that was used for the detection of antibodies against BCoV-like coronaviruses, since point mutations and deletions of the spike gene have been described in these viruses. In fact, the ELISA that was used in this study was validated only in bovine. In the absence of a validated assay for BCoV-like coronavirus antibodies, only adapted kits can be used. The validation of the ELISA is a typical issue for buffalo investigations, although according to the literature, ELISA that was designed for the bovine species may be utilized in the buffalo species (an example is represented by ELISA for the detection of bovine herpesvirus antibodies) [37]. BCoV and BCoV-like coronaviruses share a large part of the genome, up to 99.5%, as, for example, described for BuCoV, and cross-react completely (several examples of extra-species use of antigens, antibodies, or even commercial kits that were validated in bovine and used in other species have been described in the literature). Coronavirus infection in buffalo has already been demonstrated using a BCoV monoclonal antibody in several studies, as well as the cross-reactivity of BuCoV in IFA using a BCoV antiserum [4,35]. Antibodies against giraffe coronavirus react in hemagglutination with BCoV strains suggesting further evidence of cross-reactivity between BCoV and BCoV-like viruses (the same has been described also for BCoV-like coronaviruses found in camelids, which react with monoclonal antibodies prepared against BCoV antigen) [4,16]. A commercial BCoV antigen capture ELISA was used to detect antibodies against BCoV-like coronavirus in zoo ruminants in a recent study [14]. Since in vivo and in vitro experiments have shown high levels of cross-protection and cross-neutralization between BCoV and BCoV-like coronavirus, we can assume that antibodies against BCoV-like coronaviruses can still be detected using commercially available assays (pending further studies on the validation of these methods in the buffalo species) [11]. To confirm the presence of antibodies against the different CoVs, the same samples should be tested using specific serum neutralization methods for BCoV and BuCoV. 

As shown by univariate analysis, different risk factors have been associated with BCoV seropositivity. We found higher seroprevalence in adult animals (> 2 years old); these data are in line with what has been found in other studies and with the persistence of detectable anti-BCoV antibodies (described for over a year from exposure) [4,38]. Even the one and only meta-analysis study (conducted in China) supports this tendency [31].

BCoV seroprevalence was significantly higher in purchased animals (42.5%) than in animals that were born on the same farm (20.5%). There are some plausible explanations for this outcome. First, the purchased animals may have seroconverted after being exposed to a predisposing factor (transportation) for BRDC and neonatal diarrhea, in which BCoV is an etiologic factor [10,39]. A recent study found that the prevalence of BCoV (detected by real-time PCR on nasal swabs) increased from 16% to 65.1% after transportation [40]. Numerous studies in the literature describe the higher prevalence of BCoV in only certain regions or countries [41,42]. Another hypothesis involves purchasing animals from areas with higher prevalence.

Concerning the specific bovine risk factors, no differences were found between animals that were kept partly on pasture and fully indoors. A study in Sweden compared the prevalence of BCoV in organic (grazing) and traditional herds and observed no significant differences [27]. Another Swedish study found that conventional (intense) herds have a higher risk of being positive than organic herds [26]. However, as also confirmed by a meta-analysis study that was carried out in China, the animal density in the herds and the size of the farm greatly affect the seroprevalence of this infection [26,29,31]. Regarding buffalo specific risk factors, the seroprevalence was higher in animals that lived with cattle as compared to those that did not. Mixed breeding is a common practice in buffalo breeding in Italy, which increases the risk of contagion with common pathogens or allows spillover. Further research has shown coexisting with cattle as a risk factor for BCoV or BCoV-like seropositivity in sheep [32]. In both cattle and water buffalo, however, the prevalence was not affected by location, as observed by Figure 1.

This study is the first that describes the presence of detectable antibodies in buffalo in Italy and defines the seroprevalence of BCoV among cattle and buffalo on a large scale. 

Animals of different species, as well as humans, are constantly in contact with each other, increasing the risk of new and old infections. CoVs pose a persistent threat to humans and animals due to the economic damage that is caused by this virus in livestock, the genetic instability that is responsible for spillover events, and the recently demonstrated susceptibility to SARS-CoV-2 infection in cattle [35,43,44,45].

The information that was obtained in this study could significantly improve BCoV surveillance and is useful for understanding the magnitude of the infection and identifying risk factors that are involved in its transmission.

## 5. Conclusions

BCoV and BcoV-like coronaviruses are prevalent among cattle and water buffalo in southern Italy. This study not only defined regional seroprevalence in this species, but also identified some risk factors that are associated with BCoV seropositivity. This information could be extremely useful for infection control and surveillance. Although our findings are sufficient to warrant nationwide surveillance, more robust research will be required in the future, such as molecular characterization or whole genome sequencing studies to understand the genetic variations at the base of changes in the viral epidemiology. Phylogeographic and phylodynamic approaches could also be interesting to highlight reasons for epidemiological differences among countries and species.

## Figures and Tables

**Table 1:** Univariate analysis (chi-square) of common potential risk factors for bovine coronavirus seropositivity.

Factor	*n*	Positive	%	95% CI	χ^2^	*p*
Total	720	222	30.8	27.5–34.2		
Species						
Cattle	419	206	49.2	44.4–53.9		
					155.8	<0.001
Buffalo	301	16	5.3	2.8–7.8		
Age						
≤24 months	134	19	14.2	8.3–20.1		
					20.4	<0.001
>24 months	586	203	34.6	30.8–38.5		
Origin						
Born on the farm	381	78	20.5	16.4–24.5		
					39.7	<0.001
Purchased	339	144	42.5	37.2–47.7		

**Table 2:** Univariate analysis (chi-square) of potential risk factors for bovine coronavirus seropositivity in cattle.

Factor	*n*	Positive	%	95% CI	χ^2^	*p*
**Total**	419	206	49.2	44.4–53.9		
**Province**						
Avellino	112	49	43.7	34.6–52.9		
Benevento	97	53	54.6	44.7–64.5	5.2	0.157
Salerno	97	54	55.7	45.8–65.6		
Caserta	113	50	44.2	30.1–53.4		
**Stabulation**						
Partly grazed	116	56	48.3	39.2–57.4		
					0.05	0.908
Stallfed	303	150	49.5	43.9–55.1		

**Table 3:** Univariate analysis (chi-square) of potential risk factors for bovine coronavirus seropositivity in water buffalo.

Factor	*n*	Positive	%	95% CI	χ^2^	*p*
**Total**	301	16	5.3	2.8–7.8		
**Province**						
Caserta	141	7	4.9	1.4–8.5		
					0.065	0.79
Salerno	160	9	5.6	2–9.2		
**Co–living with cattle**						
Yes	119	12	10.1	4.5–15–5		
					8.9	0.003
No	182	4	2.2	0.1–4.3		

## Data Availability

Data is contained within the article or supplementary material.

## Supplementary Materials

The following supporting information can be downloaded at: https://www.mdpi.com/article/10.3390/ani13050772/s1; Table S1: Data collected on sampled ruminants.
